# Plasma WFDC2 (HE4) as a Predictive Biomarker for Clinical Outcomes in Cancer Patients Receiving Anti-PD-1 Therapy: A Pilot Study

**DOI:** 10.3390/cancers17142384

**Published:** 2025-07-18

**Authors:** Makoto Watanabe, Katsuaki Ieguchi, Takashi Shimizu, Ryotaro Ohkuma, Risako Suzuki, Emiko Mura, Nana Iriguchi, Tomoyuki Ishiguro, Yuya Hirasawa, Go Ikeda, Masahiro Shimokawa, Hirotsugu Ariizumi, Kiyoshi Yoshimura, Atsushi Horiike, Takuya Tsunoda, Mayumi Tsuji, Shinichi Kobayashi, Tatsunori Oguchi, Yuji Kiuchi, Satoshi Wada

**Affiliations:** 1 Department of Clinical Diagnostic Oncology, Clinical Research Institute for Clinical Pharmacology and Therapeutics, Showa Medical University, Tokyo 157-8577, Japan; mwata@cnt.showa-u.ac.jp (M.W.); kieguchi@med.showa-u.ac.jp (K.I.); tshimizu@med.showa-u.ac.jp (T.S.); 2 Clinical Research Institute for Clinical Pharmacology and Therapeutics, Showa Medical University, Tokyo 157-8577, Japan; kyoshim1@med.showa-u.ac.jp (K.Y.); s2koba@med.showa-u.ac.jp (S.K.); 3 Department of Pharmacology, School of Medicine, Showa Medical University, Tokyo 142-8555, Japan; risako425@med.showa-u.ac.jp (R.S.); t.oguchi@med.showa-u.ac.jp (T.O.); ykiuchi@med.showa-u.ac.jp (Y.K.); 4 Pharmacological Research Center, Showa Medical University, Tokyo 142-8555, Japan; tsujim@med.showa-u.ac.jp; 5 Division of Medical Oncology, Department of Medicine, School of Medicine, Showa Medical University, Tokyo 142-8555, Japan; rohkuma@med.showa-u.ac.jp (R.O.); emikomura@med.showa-u.ac.jp (E.M.); niriguchi@med.showa-u.ac.jp (N.I.); tomo-1496@med.showa-u.ac.jp (T.I.); h.yuya0204@med.showa-u.ac.jp (Y.H.); gou.ikeda@showa-u.ac.jp (G.I.); shimokawa.masahiro@med.showa-u.ac.jp (M.S.); ariizumi@med.showa-u.ac.jp (H.A.); horiike@med.showa-u.ac.jp (A.H.); ttsunoda@med.showa-u.ac.jp (T.T.); 6 Showa Medical University Comprehensive Cancer Information Center, Showa Medical University, Tokyo 142-8555, Japan

**Keywords:** WFDC2, immune checkpoint inhibitors, biomarker, tumor progression

## Abstract

**Background/Objectives**: Immune checkpoint inhibitors (ICIs) have revolutionized cancer therapy; however, reliable biomarkers of therapeutic efficacy remain limited. We investigated the clinical utility of plasma WFDC2 levels in patients receiving anti-PD-1 antibody treatment. **Methods**: Twenty-one patients with non-small cell lung, gastric, or bladder cancer received nivolumab or pembrolizumab. Plasma WFDC2 concentrations were measured by ELISA before ICI treatment (pre-ICI) and after two and four treatment cycles. Associations between WFDC2 expression changes and overall survival (OS), progression-free survival (PFS), and tumor progression were assessed. ROC curve analyses compared the predictive performance of WFDC2, soluble PD-L1 (sPD-L1), soluble PD-1 (sPD-1), and their combinations, with the area under the curve (AUC) evaluating predictive accuracy. **Results**: Levels of WFDC2 pre-ICI and those after two cycles were significantly higher than levels in healthy donors. However, no significant differences in WFDC2 levels were found between the time points during treatment. Greater increases in WFDC2 levels were significantly correlated with shorter OS (*p* = 0.002), shorter PFS (*p* = 0.037), and tumor progression (*p* = 0.003). ROC analysis revealed that WFDC2 achieved a higher AUC (0.700) than sPD-L1 (0.538) or sPD-1 (0.650). Combining biomarkers improved the predictive accuracy, with sPD-L1 plus WFDC2 showing the highest AUC (0.825). **Conclusions**: Serial increases in plasma WFDC2 are associated with poor clinical outcomes, highlighting its potential as a biomarker. Baseline plasma WFDC2 outperformed sPD-L1 and sPD-1 diagnostically. These findings should be interpreted as exploratory and hypothesis-generating, requiring confirmation in larger, tumor-specific cohorts with multivariate adjustment. WFDC2 represents a promising minimally invasive biomarker for the early identification of patients unlikely to benefit from ICI therapy.

## Simple Summary

Immune checkpoint inhibitors (ICIs) have transformed cancer therapy. However, selecting patients who will benefit from ICIs remains challenging. In this exploratory study, we evaluated plasma WFDC2 (HE4) levels as potential dynamic biomarkers in patients treated with anti-PD-1 antibodies. WFDC2 levels increased significantly after treatment initiation, and greater increases correlated with worse overall survival, progression-free survival, and tumor progression. Compared with soluble PD-L1 and PD-1, WFDC2 demonstrated higher predictive performance in receiver operating characteristic (ROC) analyses. Combining WFDC2 with other biomarkers enhanced prediction accuracy. These findings suggest that monitoring WFDC2 levels during treatment could enable the early identification of patients unlikely to respond to ICIs and support personalized therapy decisions. However, larger studies are needed to validate its utility across different cancer types and treatment settings.

## 1. Introduction

Immune checkpoint inhibitors (ICIs), particularly antibodies targeting programmed cell death protein 1 (PD-1) and its ligand PD-L1, have revolutionized cancer treatment by restoring T cell-mediated antitumor immunity [1,2,3]. These agents have become the mainstay of treatment for several malignancies, including non-small cell lung cancer (NSCLC), gastric cancer, melanoma, and urothelial carcinoma [4,5]. Despite their remarkable therapeutic potential, a significant proportion of patients fail to respond to ICIs or experience disease progression following an initial response, highlighting the urgent need for predictive and pharmacodynamic biomarkers [6,7,8].

Currently, the expression of PD-L1 in tumor tissues, evaluated by immunohistochemistry, is the most widely used biomarker for predicting the response to PD-1/PD-L1 blockade [9]. However, this approach has several limitations. Tumor PD-L1 expression is heterogeneous, temporally dynamic, and influenced by previous treatments [10]. Moreover, tissue biopsies are invasive and not always feasible, particularly for repeated monitoring. Therefore, circulating biomarkers that reflect the tumor immune microenvironment in a minimally invasive manner are highly desirable [11,12].

Among these markers, the soluble forms of PD-1 (sPD-1) and PD-L1 (sPD-L1) are promising candidates. These molecules are generated either through alternative mRNA splicing or the proteolytic shedding of membrane-bound receptors and ligands and can be readily detected in the bloodstream using enzyme-linked immunosorbent assays (ELISAs) [13]. Increasing evidence suggests that circulating sPD-1 and sPD-L1 levels are dynamically regulated during ICI therapy and may have clinical relevance in predicting response and prognosis [14].

For example, increased plasma sPD-1 concentrations are associated with disease progression in patients treated with anti-PD-1 antibodies [15]. Conversely, decreased plasma sPD-L1 levels have been shown to correlate with tumor regression in patients with lung and gastric cancers undergoing ICI therapy [16]. However, a recent meta-analysis of individual patient data for advanced NSCLC found no significant association between sPD-L1 dynamics and survival outcomes, suggesting that the prognostic value of sPD-L1 may be context-dependent and influenced by factors such as an assay platform, the timing of measurement, and patient population [17]. This discrepancy highlights the need for further investigation to clarify the clinical relevance of sPD-L1 and other soluble markers in diverse patient cohorts.

WFDC2 (whey acidic protein four-disulfide core domain 2, also known as human epididymis protein 4 [HE4]), initially recognized as a biomarker of ovarian cancer [18,19], has recently attracted considerable attention because of its elevated expression in lung adenocarcinoma (LUAD) and other malignancies. In LUAD, WFDC2 protein and mRNA levels are significantly higher in tumor tissues than in normal tissues, and high WFDC2 expression correlates with improved overall survival (OS) [20,21]. Moreover, WFDC2 expression is associated with the TP53 mutation status and is involved in cell cycle regulation. Low WFDC2 expression correlates with increased tumor mutational burden and neoantigen load and negatively correlates with immune-related gene expression. Importantly, high WFDC2 expression was more frequently observed in patients with low PD-1 mRNA expression, suggesting a potential role for WFDC2 in modulating the immune checkpoint inhibitor response in LUAD [22].

Although these associations point to a role for WFDC2 in tumor biology, particularly in modulating immune responses, its function during ICI therapy remains unclear. Preclinical studies suggest involvement in extracellular matrix remodeling and immune suppression, but clinical validation is lacking.

Given the variability in tumor biology across cancer types, including NSCLC, gastric, and bladder cancers, biomarker dynamics may differ significantly. Exploratory evaluation of circulating markers in diverse cohorts may provide hypotheses for subsequent mechanistic or tumor-specific validation.

In this pilot study, we longitudinally examined early changes in serum sPD-1, sPD-L1, and WFDC2 levels in patients with advanced cancer receiving anti-PD-1 antibodies. Our goal was to assess whether WFDC2 dynamics might complement established soluble immune markers and to explore their association with treatment outcomes. While definitive conclusions cannot be drawn due to the limited sample size, our findings should be explicitly framed as exploratory and require validation in larger, independent cohorts. Nonetheless, our findings provide a basis for future hypothesis-driven research.

## 2. Materials and Methods

### 2.1. Patient Information and Plasma Sample Collection

We retrospectively analyzed the clinical data and archived plasma samples from 21 patients who received anti-PD-1 antibody therapy at Showa Medical University Hospital between January 2017 and April 2019. Plasma samples were obtained from six healthy volunteers as controls. The study cohort included 11 patients with first-line or previously treated NSCLC, nine with gastric cancer, and one with bladder cancer. According to the Union for International Cancer Control Tumor–Node–Metastasis classification (7th edition), 14 and 7 patients were diagnosed with stage IV and III disease, respectively. Some patients had experienced recurrent disease following curative surgery or chemoradiotherapy. Patients were treated with either nivolumab (240 mg intravenously every 2 weeks) or pembrolizumab (200 mg intravenously every 3 weeks); five patients with NSCLC and nine with gastric cancer received nivolumab, whereas six patients with NSCLC and one with bladder cancer received pembrolizumab. The clinicopathological characteristics of eligible patients were described in Table S1.

To investigate the association between plasma WFDC2 concentrations and clinical responses to PD-1 blockade therapy in cancer patients, plasma samples were collected at three time points: before therapy initiation (baseline), after two treatment cycles, and after four treatment cycles. Plasma was isolated using a BD P100 Blood Collection System (366422, BD Biosciences, Franklin Lakes, NJ, USA) according to the manufacturer’s instructions. The obtained plasma was aliquoted and stored at −80 °C until analysis.

Relative changes in sPD-1 and sPD-L1 levels were calculated by dividing post-treatment values (after two or four cycles) by their respective baseline values. Tumor response was assessed according to the Response Evaluation Criteria in Solid Tumors (RECIST), version 1.1, using computed tomography performed before treatment and after four cycles. The change in tumor size was calculated as the percentage change in the sum of the longest diameters of target lesions from baseline. PFS was defined as the time from initiation of anti-PD-1 therapy to disease progression or death from any cause. OS was defined as the time from diagnosis to the date of last follow-up or death.

### 2.2. Quantification of Plasma WFDC2 by ELISA

Plasma concentrations of WFDC2 were quantified using the Human HE4/WFDC2 DuoSet ELISA Development System (DY6274, R&D Systems, Minneapolis, MN, USA), following the manufacturer’s protocol. Briefly, 96-well plates were coated with anti-WFDC2 capture antibodies overnight. The following day, the plates were washed with 0.05% Tween 20 in phosphate-buffered saline (PBS) and blocked for 1 h with 1% bovine serum albumin (BSA) in PBS. Plasma samples were centrifuged at 3000 rpm for 10 min at 4 °C, and the resulting supernatants were diluted 1:20 in PBS containing 1% BSA. After washing, 100 µL of each diluted sample was added to the wells and incubated for 2 h. Subsequent steps included incubation with biotin-labeled detection antibodies, streptavidin–horseradish peroxidase, and tetramethylbenzidine substrate. The reaction was stopped with 2 N sulfuric acid, and the absorbance was read at 450 nm with a reference wavelength of 570 nm using a BioTek Synergy HTX Multimode Reader and analyzed with Gen5 software. All procedures were conducted at ambient temperature (20–25 °C) unless otherwise noted. Detailed protocols for measuring sPD-L1 and sPD-1 levels have been described previously in reference [13,14].

### 2.3. Statistical Analysis

Statistical analysis was performed using JMP Pro 17.0 (SAS Institute Inc., Cary, NC, USA). The non-parametric Steel–Dwass test was used for comparisons among three or more groups, including healthy donors and each treatment time point during ICI therapy. Survival probabilities were estimated using the Kaplan–Meier method and compared using the log-rank test. Univariate Cox proportional hazards regression was used to assess the associations with survival. Receiver operating characteristic (ROC) curves were generated based on stable disease/progressive disease (SD/PD), and complete/partial response (CR/PR) was defined by best overall response (BOR). Binary logistic regression was applied to both univariate and multivariate analyses to evaluate the predictive value of each biomarker and its combinations. All statistical tests were two-sided, and *p*-values < 0.05 were considered statistically significant.

## 3. Results

### 3.1. Association Between Tumor WFDC2 mRNA Expression and Survival Outcomes in Patients Undergoing Anti-PD-1 Therapy

**Figure 1:**
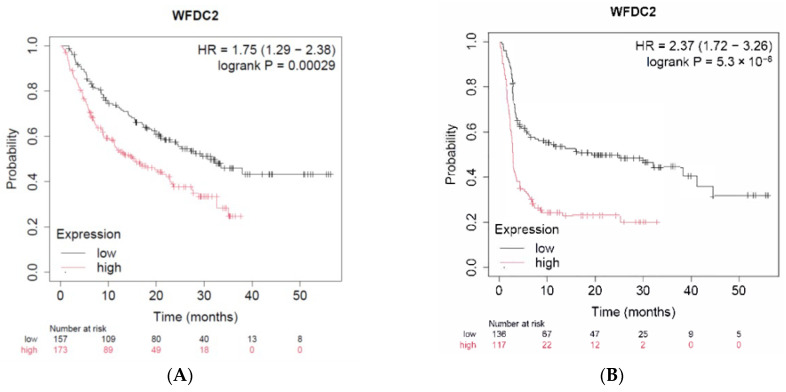
**Association between tumor WFDC2 mRNA expression and survival outcomes in patients undergoing anti-PD-1 therapy.** Two patient cohorts stratified by high versus low WFDC2 mRNA expression are compared using Kaplan–Meier survival analysis. Hazard ratios with 95% confidence intervals and log-rank *p*-values are calculated. (**A**) OS curves. (**B**) PFS curves. The number of patients at risk is indicated below each time point on the respective survival plots.

To assess the prognostic significance of tumor WFDC2 mRNA expression in patients receiving anti-PD-1 ICIs, Kaplan–Meier survival analyses were performed using data from the Kaplan–Meier Plotter database, which integrates gene expression and clinical outcomes from multiple public datasets [23]. The analyzed cohort comprised 520 patients with diverse cancer types treated with either nivolumab or pembrolizumab. Tumor RNA samples were collected before therapy initiation. Patients were stratified into high and low WFDC2 expression groups based on the platform’s automated cutoff. In the OS analysis (Figure 1A), high WFDC2 expression was significantly associated with poorer prognosis (hazard ratio [HR] = 1.75, 95% confidence interval [CI]: 1.29–2.38; log-rank *p* = 0.00029). Similarly, PFS analysis (Figure 1B) demonstrated significantly reduced survival in the high-expression group (HR = 2.37, 95% CI: 1.72–3.26; log-rank *p* = 5.3 × 10^−8^). The number of patients at risk at each time point is indicated by Kaplan–Meier curves. No further stratification by cancer type, demographics, or treatment regimen was performed. These results indicated that elevated tumor WFDC2 mRNA expression prior to anti-PD-1 therapy correlated with unfavorable survival outcomes.

### 3.2. Temporal Changes in Plasma sPD-L1, sPD-1, and WFDC2 Levels During Anti-PD-1 Therapy

The plasma concentrations of soluble PD-L1 (sPD-L1), soluble PD-1 (sPD-1), and WFDC2 were measured using ELISA at four time points: healthy controls and cancer patients before ICI treatment (pre-ICI), after two cycles of anti-PD-1 therapy (after two cycles), and after four cycles (after four cycles). This longitudinal analysis enabled the evaluation of biomarker dynamics during treatment and comparison with baseline healthy levels.

**Figure 2:**
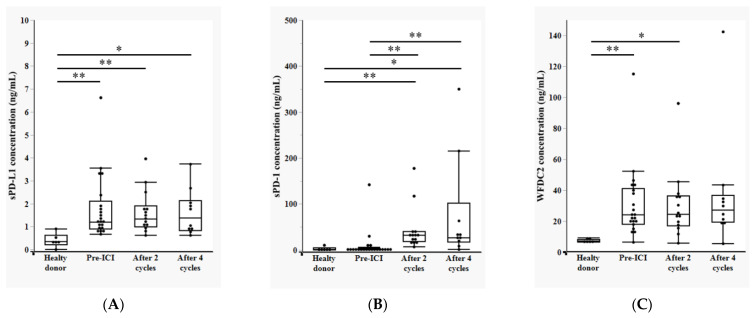
**Temporal changes in plasma levels of sPD-L1, sPD-1, and WFDC2 during anti-PD-1 therapy:** (**A**) Plasma concentrations of soluble PD-L1 (sPD-L1) measured by ELISA at three time points: healthy controls (*n* = 6), cancer patients before immune checkpoint inhibitor treatment (pre-ICI) (*n* = 21), after two cycles of anti-PD-1 therapy (after 2 cycles) (*n* = 14), and after four cycles (after 4 cycles) (*n* = 10). (**B**) Plasma levels of soluble PD-1 (sPD-1) at the same time points. (**C**) Plasma WFDC2 levels at the same time points. Statistical comparisons among groups were performed using the Steel–Dwass test for multiple comparisons. Statistical significance is indicated as * *p* < 0.05, ** *p* < 0.01.

sPD-L1 levels were significantly elevated in all patient groups (pre-ICI, after two cycles, and after four cycles) compared with healthy controls, with no significant differences observed among the patient groups, indicating sustained elevation throughout treatment (Figure 2A). Conversely, sPD-1 levels in the pre-ICI group were comparable to those in healthy controls but increased significantly after two and four cycles compared to both healthy controls and pre-ICI levels, suggesting treatment-induced elevation of circulating sPD-1 (Figure 2B). WFDC2 levels displayed a pattern similar to that of sPD-L1, being significantly elevated in the pre-ICI and after-two-cycles groups compared with healthy controls. However, by the fourth cycle, WFDC2 levels were no longer significantly different from those in healthy individuals, suggesting potential normalization during continued therapy (Figure 2C).

Collectively, these findings demonstrate distinct longitudinal profiles for plasma sPD-L1, sPD-1, and WFDC2 levels during anti-PD-1 treatment, potentially reflecting the differential regulation and functional roles of these biomarkers in the context of immunotherapy.

### 3.3. Correlation Between Plasma Biomarker Dynamics and Clinical Outcomes During ICI Therapy

To explore the clinical relevance of plasma biomarker dynamics during ICI treatment, associations between absolute plasma concentrations of sPD-L1, sPD-1, and WFDC2 at each time point (pre-ICI, after two cycles, and after four cycles) and clinical outcomes—including OS, PFS, and changes in tumor size—were analyzed using Spearman’s rank correlation. No significant correlations were observed between absolute biomarker levels and clinical outcomes at any time point.

**Figure 3:**
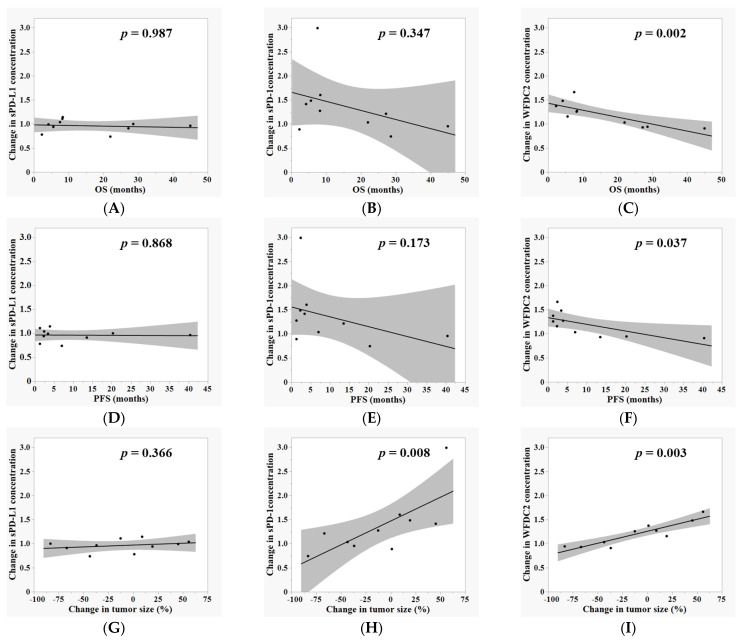
**Correlation between plasma biomarker dynamics and clinical outcomes during ICI therapy.** Spearman correlation analyses are conducted to evaluate associations between fold changes in plasma biomarker levels from after two to after four cycles of ICI therapy and clinical outcomes, including survival and tumor response. Correlations are shown between fold changes in sPD-L1 (**A**), sPD-1 (**B**), and WFDC2 (**C**) and OS; sPD-L1 (**D**), sPD-1 (**E**), and WFDC2 (**F**) and PFS; and sPD-L1 (**G**), sPD-1 (**H**), and WFDC2 (**I**) and change in tumor size. Fold changes are calculated relative to levels after two cycles of treatment. A *p*-value < 0.05 is considered statistically significant.

Subsequently, fold changes in biomarker levels between time points (pre-ICI to after two cycles, after two to four cycles, and pre-ICI to after four cycles) were evaluated for correlations with clinical outcomes. For sPD-L1, the fold change from pre-ICI to after four cycles correlated positively with change in tumor size (ρ = 0.661, *p* = 0.038), indicating that increased sPD-L1 levels were associated with poorer tumor response. However, fold changes in sPD-L1 levels from two to four cycles showed no significant correlation with OS, PFS, or change in tumor size (Figure 3A–C). For sPD-1, fold changes from after two to after four cycles correlated positively with change in tumor size (ρ = 0.782, *p* = 0.008), suggesting that increasing sPD-1 levels were also associated with poor tumor response; however, no significant correlations were observed with OS or PFS (Figure 3D–F). WFDC2 demonstrated the strongest associations: fold increases from after two to after four cycles correlated positively with change in tumor size (ρ = 0.830, *p* = 0.003) and inversely with OS (ρ = −0.830, *p* = 0.003) and PFS (ρ = −0.711, *p* = 0.021), indicating that increasing WFDC2 levels reflect disease progression and predict poorer survival (Figure 3G–I).

These data collectively suggest that, among the three biomarkers, dynamic changes in WFDC2, alongside sPD-1 and sPD-L1 (to a lesser extent), are significantly associated with tumor response and survival outcomes during anti-PD-1 therapy.

### 3.4. Survival Analysis Based on Baseline Pre-ICI Plasma Levels of sPD-L1, sPD-1, and WFDC2

**Figure 4:**
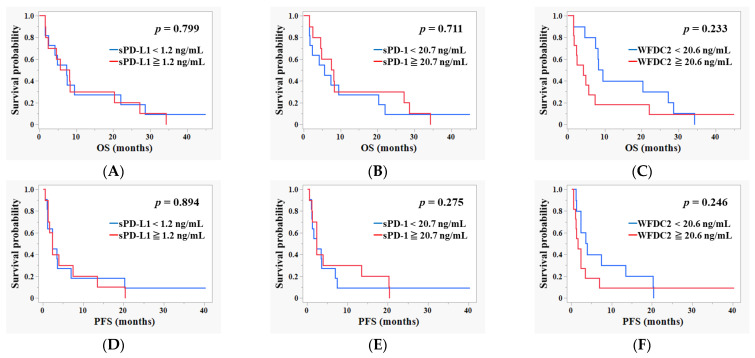
**Survival analysis based on baseline pre-ICI plasma levels of sPD-L1, sPD-1, and WFDC2.** Kaplan–Meier curves showing OS and PFS stratified by plasma concentrations of sPD-L1 (**A**,**D**), sPD-1 (**B**,**E**), and WFDC2 (**C**,**F**) in cancer patients undergoing ICI therapy. Panels (**A**–**C**) show OS, and panels (**D**–**F**) show PFS. Patients are divided into high and low groups based on median biomarker levels. Statistical significance is assessed by a log-rank test. *p* < 0.05 is considered statistically significant.

To determine the prognostic potential of baseline plasma biomarkers in patients with cancer undergoing ICI therapy, survival analyses were performed by stratifying patients into high and low groups based on the median plasma concentrations of sPD-L1, sPD-1, and WFDC2. The Kaplan–Meier curves for OS and PFS revealed no statistically significant differences between the groups for any biomarker (Figure 4A–F). Baseline plasma levels of sPD-L1, sPD-1, and WFDC2 were not significantly associated with altered survival outcomes.

### 3.5. Evaluation of the Diagnostic Performance of Baseline Plasma Biomarkers for Predicting BOR to ICI

Logistic regression analysis was performed to evaluate the association between baseline WFDC2 levels and the BOR to immune checkpoint inhibitor therapy. For this analysis, the BOR was treated as a binary variable, classifying patients with CR or PR as responders and those with SD or PD as non-responders.

**Figure 5:**
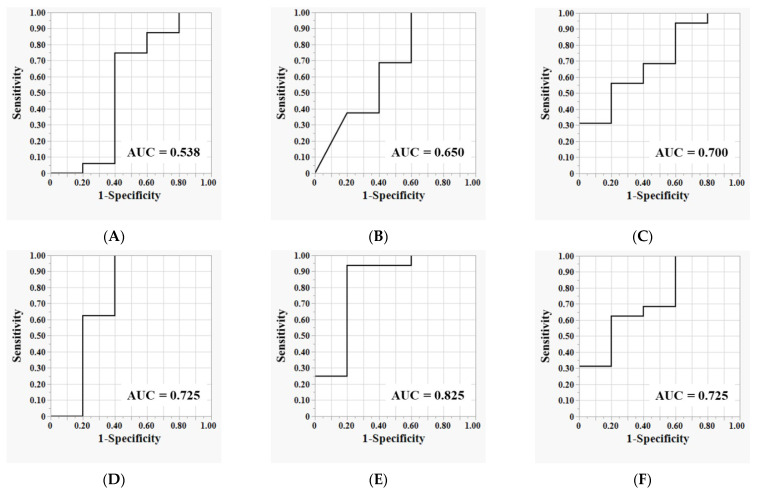
**Evaluation of the diagnostic performance of baseline plasma biomarkers for predicting BOR to ICI.** ROC curves are generated using binary logistic regression models to distinguish non-responders (patients with SD or PD) from responders (patients with CR or PR). Individual ROC curves are shown for (**A**) sPD-L1, (**B**) sPD-1, and (**C**) WFDC2. Combined biomarker models include (**D**) sPD-L1 plus sPD-1, (**E**) sPD-L1 plus WFDC2, and (**F**) sPD-1 plus WFDC2. AUC values are indicated within each plot.

The predictive capacities of baseline plasma sPD-L1, sPD-1, and WFDC2 levels for the BOR were assessed using ROC curve analyses. Individually, sPD-L1 showed limited discriminative ability (area under the curve [AUC]: 0.538; 95% confidence interval [CI]: 0.33–0.75) (Figure 5A), while sPD-1 demonstrated moderate predictive value (AUC: 0.650; 95% CI: 0.45–0.85) (Figure 5B). Among the single biomarkers, WFDC2 exhibited superior diagnostic performance (AUC: 0.700; 95% CI: 0.50–0.90) (Figure 5C). Importantly, combining biomarkers improved the predictive accuracy. The combination of sPD-L1 and sPD-1 reached an AUC of 0.725 (95% CI: 0.53–0.92) (Figure 5D). Notably, the combination of sPD-L1 and WFDC2 yielded the highest AUC (0.825; 95% CI: 0.66–0.99) (Figure 5E), indicating a strong synergistic effect. The combination of sPD-1 and WFDC2 also achieved an AUC of 0.725 (95% CI: 0.53–0.92) (Figure 5F), demonstrating a similar enhancement in predictive capability.

These results indicate that the baseline plasma WFDC2 level serves as a relatively robust standalone biomarker for predicting BOR to ICI therapy. Furthermore, when combined with the soluble immune checkpoint-related biomarkers sPD-L1 and sPD-1, WFDC2 provides additive and complementary diagnostic value, significantly improving prediction accuracy. This highlights the potential clinical utility of incorporating WFDC2 into multiplex biomarker panels to enhance patient stratification and to optimize therapeutic decision making in the context of ICI treatment.

## 4. Discussion

In this study, we exploratorily evaluated plasma WFDC2 levels during anti-PD-1 antibody therapy in patients with NSCLC, gastric cancer, and bladder cancer. Notably, greater increases in WFDC2 levels were associated with tumor progression, shorter OS, and shorter PFS. These findings were consistently observed across multiple cancer types, suggesting that WFDC2 dynamics may serve as a common biomarker for ICI response. However, given the small sample size and tumor heterogeneity, these findings should be interpreted as hypothesis-generating. These findings suggest that dynamic changes in circulating WFDC2 may reflect biological processes underlying resistance to ICIs and that WFDC2 could serve as a novel blood-based biomarker for the early identification of non-responders.

WFDC2 is a secreted glycoprotein that was initially established as a diagnostic biomarker for ovarian cancer [24]. It has been shown to be overexpressed in various solid tumors, including those of the lungs, pancreas, and gastrointestinal tract [25,26]. Accumulating evidence indicates that WFDC2 may actively contribute to malignancy by promoting proliferation, migration, and immune evasion [27,28,29,30]. However, its role in modulating ICI response remains poorly understood. Our study is among the first to longitudinally evaluate plasma WFDC2 levels during immunotherapy, revealing that rising levels may serve as a surrogate marker associated with ICI resistance. This suggests that WFDC2 may be leveraged not only for baseline risk stratification but also for on-treatment monitoring to optimize therapeutic decisions.

A recent single-cell RNA sequencing study of NSCLC supports the involvement of WFDC2 in immune escape [31]. In this study, tumor samples from eight lung adenocarcinoma patients were grouped based on PD-L1 expression. Among the 58,810 cells analyzed, one tumor cell cluster characterized by high WFDC2 expression was identified. WFDC2, along with NAPSA and MUC1, was upregulated in PD-L1–positive tumor cells, suggesting its potential role in immune evasion [31]. This cellular evidence provides mechanistic insights into the potential role of WFDC2 in shaping the immunosuppressive tumor microenvironment. These findings suggest that WFDC2 may influence immune resistance through pathways independent of the PD-1/PD-L1 axis.

These findings reinforce the hypothesis that WFDC2 not only serves as a biomarker but also participates in tumor immunobiology. As a non-immune biomarker, WFDC2 may offer insights into the tumor microenvironment beyond those provided by conventional immune markers [32]. It has been implicated in extracellular matrix remodeling and in regulating cytokines involved in immune suppression [33,34]. The upregulation of WFDC2 during ICI therapy may reflect the activation of resistance pathways independent of the PD-1/PD-L1 axis or may signal tumor adaptation processes, such as epithelial–mesenchymal transition, increased tumor burden, or stromal remodeling, all of which have been associated with immunotherapy resistance [35,36]. These observations raise the hypothesis that WFDC2 may exert its immunomodulatory effects through defined signaling pathways. Further functional studies are needed to clarify whether WFDC2 directly promotes these resistance mechanisms or serves as a downstream marker of broader tumor adaptation.

A key strength of this study lies in the serial measurement of plasma samples at predefined time points, enabling the exploratory assessment of WFDC2 dynamics during ICI therapy. WFDC2 levels were significantly higher in patients than in healthy donors, both at baseline and after two treatment cycles, whereas no significant change was observed between these time points. These findings suggest that WFDC2 levels are elevated in patients with cancer before treatment and may increase further early during therapy. Notably, the degree of elevation after two cycles was significantly associated with poor outcomes, including disease progression and shorter OS and PFS. This temporal relationship highlights the potential clinical utility of WFDC2 for the prediction of treatment resistance early on, which could inform therapeutic adjustments before imaging-based progression becomes evident. Interestingly, WFDC2 levels after cycle 4 were not significantly different from baseline levels, which may indicate that, in some patients, WFDC2 levels stabilize or decline after initial elevation, possibly reflecting immune activation or reduced tumor burden in responders. This transient normalization contrasts with previous reports on soluble PD-L1 (sPD-L1) dynamics [17], where sustained changes were observed. These discrepancies may arise from differences in assay sensitivity, patient selection, and cancer types studied. Careful consideration of these factors is crucial when interpreting and comparing biomarker kinetics across studies. The transient versus sustained patterns of WFDC2 elevation might reflect distinct biological responses, where transient increases could indicate effective immune activation or tumor control, while sustained elevation may signal ongoing resistance to ICI. The integration of WFDC2 dynamics with radiographic and immune cell profiling (e.g., CD8^+^ T cells) may help contextualize these observations.

However, this study has some limitations. First, the sample size was small (*n* = 21) and heterogeneous in terms of tumor type and ICI agent used (nivolumab or pembrolizumab). Although reflective of real-world practice, larger and more homogeneous cohorts are required for validation. Pooling across tumor types and ICI regimens may have masked disease- or drug-specific biomarker patterns. Furthermore, WFDC2 levels can be influenced by renal dysfunction and smoking, as shown in previous reports [37,38]. These are important confounders, and studies with age-, sex-, renal function-, and smoking status-matched controls will be essential to better isolate the predictive value of WFDC2 and should be considered in future research. Second, although we focused on the WFDC2 protein levels, the mechanisms underlying its increase during therapy remain unclear. Potential mechanistic pathways include WFDC2’s interaction with immune checkpoints such as TIM-3, which may promote T cell exhaustion and contribute to resistance to ICIs. Additionally, WFDC2 has been implicated in activating the TGF-β signaling pathway, a key mediator of fibrosis and immune suppression in the tumor microenvironment [39]. Experimental approaches such as co-immunoprecipitation, functional assays with TIM-3 blockade, and the evaluation of TGF-β pathway activity could clarify these interactions. Elucidating these mechanisms will be essential to understand whether WFDC2 directly drives immune resistance or serves as a biomarker of broader tumor adaptation.

Further studies are needed to elucidate the biological role of WFDC2 in ICI resistance. We also acknowledge the potential bias introduced by excluding patients who experienced rapid disease progression and thus lacked post-treatment samples. The absence of multivariate adjustments for confounders such as tumor stage, prior therapies, and inflammation is a limitation related to the small sample size, and future studies should address this using appropriate modeling to better clarify the independent role of WFDC2. Taken together, these limitations highlight the exploratory nature of our findings and the necessity for future larger prospective studies to validate and extend these results. Finally, comparisons with other emerging markers could help define the specificity and relative value of WFDC2 in clinical settings. Our analysis combining WFDC2 with sPD-L1 and sPD-1 in a small cohort may risk overfitting; thus, external validation and cross-validation are essential before clinical application. Future prospective studies incorporating multiomics profiling and functional assays may help uncover the mechanistic basis and predictive power of WFDC2.

## 5. Conclusions

In this exploratory study, serial progressive increases in plasma WFDC2 levels have been associated with poor clinical outcomes, indicating its potential as a dynamic biomarker. Baseline WFDC2 levels showed better diagnostic accuracy than sPD-L1 and sPD-1, and combining WFDC2 with sPD-L1 further improved predictive accuracy. These findings emphasize that WFDC2 is a promising noninvasive biomarker for the early identification of patients who are unlikely to benefit from ICI therapy. Nevertheless, given the limited sample size and tumor heterogeneity, these findings warrant cautious interpretation. Additional validation in larger, prospective cohorts stratified by cancer type is essential to establish the clinical applicability of WFDC2 and to clarify its underlying mechanisms related to ICI resistance.

## Data Availability

The dataset is available upon request from the authors.

## Supplementary Materials

The following supporting information can be downloaded at: https://www.mdpi.com/article/10.3390/cancers17142384/s1, Table S1: The clinicopathological characteristics of eligible patients.

## Abbreviations

The following abbreviations are used in this manuscript:

ICI	Immune checkpoint inhibitor
WFDC2	Whey acidic protein four-disulfide core domain 2
HE4	Human Epididymis Protein 4
OS	Overall survival
PFS	Progression-free survival
ROC	Receiver operating characteristic
PD-L1	Programmed death-ligand 1
PD-1	Programmed cell death protein 1
sPD-L1	Soluble programmed death-ligand 1
sPD-1	Soluble programmed cell death protein 1
NSCLC	Non-small cell lung cancer
ELISA	Enzyme-linked immunosorbent assay
BOR	Best overall response
CR	Complete response
PR	Partial response
SD	Stable disease
PD	Progressive disease
AUC	Area under the curve
RECIST	Response Evaluation Criteria in Solid Tumors
HR	Hazard ratio
CI	Confidence interval
OR	Odds ratio
